# Effects of an individualized home-based unsupervised aerobic training on body composition and physiological parameters in obese adults are independent of gender

**DOI:** 10.1007/s40618-017-0771-2

**Published:** 2017-10-28

**Authors:** G. P. Emerenziani, M. C. Gallotta, S. Migliaccio, D. Ferrari, E. A. Greco, F. J. Saavedra, S. Iazzoni, A. Aversa, L. M. Donini, A. Lenzi, C. Baldari, L. Guidetti

**Affiliations:** 1 Department of Experimental and Clinical Medicine, University of Magna Græcia of Catanzaro, Catanzaro, Italy; 2 Section of Health Sciences, Department of Movement, Human and Health Sciences, University of Rome “Foro Italico”, Piazza Lauro De Bosis 6, 00135 Rome, Italy; 3 Section of Medical Pathophysiology, Food Science and Endocrinology, Department of Experimental Medicine, Sapienza University of Rome, Rome, Italy; 4 Research Centre for Sports Sciences, Health and Human Development Sport Sciences Department, University of Trás-os-Montes e Alto Douro, Vila Real, Portugal

**Keywords:** Obesity, Physical activity, Cardio fitness, Aerobic training, Gender differences

## Abstract

**Purpose:**

Evaluation of the effects of an individualized home-based unsupervised aerobic training on body composition, physical and physiological parameters in female and male obese adults.

**Methods:**

Two hundred and twenty obese adults (age 47.9 ± 12.4 years; BMI 38.0 ± 7.2 kg/m^2^) entered the 4-month training program. Body composition, physiological and functional capacities were assessed pre- and post-intervention. All subjects were requested to perform unsupervised aerobic training with the intensity based on heart rate, walking speed and OMNI-RPE score corresponding to the individual ventilatory threshold for at least 5 days/week.

**Results:**

After 4-month study period, 40% of patients completed the protocol, 24% had high compliance (HC) (exercise ≥ 3 days/week), while 16% had low compliance (LC) to exercise prescription (exercise < than 3 days/week). In HC group, a significant improvement of body composition variables after training was performed. Moreover, oxygen uptake and metabolic equivalent at peak significantly increased after training. Six-minute walking test (6MWT) distance significantly increased while heart rate during 6MWT was significantly lower after training. No significant differences were found in LC group between pre- and post-intervention in all variables. Interestingly, gender does not influence the effects of training.

**Conclusions:**

Our results indicate that subjects, independent of gender, with high compliance to the aerobic training based on a new individualized method can achieve a significant reduction in weight loss and also an improvement in physical and physiological parameters. This innovative personalized prescription could be a valuable tool for exercise physiologist, endocrinologists, and nutritionists to approach and correct life style of obese subjects.

## Introduction

Obesity is a multifactorial pathological condition characterized by an excess of adipose tissue in the human body (> 35% in women > 25% in men) leading to a significant increased risk to develop metabolic chronic diseases [1]. Obesity is a predictor of metabolic syndrome, described as a clustering of risk factors (e.g., central obesity, type 2 diabetes mellitus) becoming one of the most common causes of death worldwide [2].

It is well known that regular physical activity (PA) provides health benefits and it is considered an essential component of primary and secondary prevention for most of metabolic syndrome-related pathologies [3]. However, physical inactivity and sedentary behavior are considered a global health problem. Half of subjects who begin exercise programs to increase physical activity quit within few months [4]. Supervision has been shown to be effective in helping adults adhere to an exercise plan [5–7] but this approach often requires a center-based approach, which it might be not widely accessible in obese population for financial, logistic barriers and dependence on caregivers. Therefore, it seems necessary to implement more feasible approaches of exercise training so that a greater number of patients could benefit from regular physical exercise in an almost daily routine.

The role of exercise intensity on physical training adherence could play a fundamental role [8, 9] in regards of the effects obtained and it should be established to achieve positive physiological effects and decreased injury risk, resulting in an improved adherence to physical activity. Considering the individual exercise capacity, the use of individual ventilatory threshold (IVT) and rate of perceived exertion (RPE) scale have been used in unfit populations to prescribe exercise intensity [10–14]. In particular, IVT is considered a useful submaximal breakpoint for optimal moderate exercise intensity prescription in T2DM patients and obese subjects [15–17] in both genders. The work load corresponding to these physiological events is termed as “ventilatory threshold 1” or “aerobic threshold” [13]; thus, the term “individual ventilatory threshold” (IVT) will be used in the manuscript.

This method could be more easily applied in a laboratory or in supervised conditions due to the controlled environment (the presence of trainer and the possibility to control the body and environmental variables such as heart rate and walking speed).

Conversely, the use of IVT for unsupervised moderate training to modify the subjects’ lifestyle is difficult to apply. For instance, the use of walking speed corresponding to IVT could not be applied alone if the walking track is in an urban or rural setting due to the different characteristics of the track (positive or negative slopes). Moreover, maintenance of a heart rate (HR) intensity similar to the HR corresponding to IVT during training is a difficult task. Due to these reasons, it would be useful to apply a new method that includes more than one variable which could lead the subjects to control the exercise intensity corresponding to their IVT.

Rate of perceived exertion (RPE) is defined as the individual intensity of effort, strain, and/or fatigue that is experienced during physical exercise, and it can be used as a valid tool to assess the individuals’ perceptions of effort during exercise [18]. The RPE represents an inexpensive tool for monitoring exercise intensity in unfit populations [10] and it is suggested by the American College of Sports Medicine to modulate and refine the prescription of exercise intensity [19–21]. OMNI scales of perceived exertion (RPE-OMNI) were validated for adult and elderly populations [22]. RPE-OMNI scale is intuitive and simple to understand and it is valid and reliable for monitoring exercise intensity.

Walking is a traditional exercise type, which promotes healthy lifestyle and fitness in obese subjects but, due to lack of controlling exercise intensity, obese individuals are often unable to reach their training objectives [23]. Therefore, both IVT and the RPE-OMNI-Walk/Run Scale variables could be very useful tools if used together, to prescribe home-based unsupervised walking exercise. While literature highlights the positive effects of supervised aerobic training, there are no studies showing evidence on the effects of home-based unsupervised aerobic training based upon HR, walking speed, and RPE-OMNI-Walk/Run Scale corresponding to IVT in obese subjects.

Therefore, aims of the study were: (1) to evaluate whether a 4-month home-based unsupervised aerobic training program, based upon gait speed, HR, and RPE-OMNI score corresponding to IVT, exerted positive effects on body composition, physical and physiological capacity and (2) to verify the adherence to an individualized exercise program in a real life conditions in female and male obese subjects.

## Materials and methods

### Participants

Two hundred and twenty (48 males, 172 females) obese adults (age 48.1 ± 12.3 years; BMI 38.0 ± 6.5 kg/m^2^) were selected from a large group of patients admitted to the Day Hospital of the Department of Experimental Medicine, Section of Medical Pathophysiology, Food Science and Endocrinology, Policlinico Umberto I, “Sapienza” University of Rome. Subjects underwent clinical examination to rule out any contraindications to physical activity (PA). The inclusion criteria were adult age (> 30 year) and a BMI ≥ 30 or a fat mass ≥ 35% in women and ≥ 25% in men [1]. Body composition was evaluated by hand–foot bioelectrical impedance method (Tanita BC 601, Tokyo, Japan) [24]. The descriptive characteristics of the subjects are summarized in Table 1. All subjects were sedentary and they had not been previously engaged in regular physical exercise. All subjects provided a written informed consent before participating in the study. All procedures were approved by the Local Ethics Committee, which was in accordance with principles outlined in the Declaration of Helsinki.

**Table 1:** Comparison of anthropometric and body composition parameters between males and females at the beginning of the study (males *n* = 48; females *n* = 172)

	Males	Females
Age (years)	49.3 ± 12.0	47.8 ± 12.3
Height (m)	1.73 ± 0.06	1.61 ± 0.07*
Weight (kg)	114.1 ± 18.9	98.3 ± 19.6*
BMI (kg/m^2^)	37.8 ± 5.8	38.0 ± 6.7
FM (%)	36.3 ± 5.2	45.8 ± 5.2*
FFM (kg)	73.0 ± 10.2	52.1 ± 7.8*
FFM (%)	64.3 ± 7.5	53.4 ± 5.4*

*BMI* body mass index, *FFM* fat-free mass, *FM* fat mass

*p < 0.01 vs. males

### Experimental Design

**Fig. 1:**
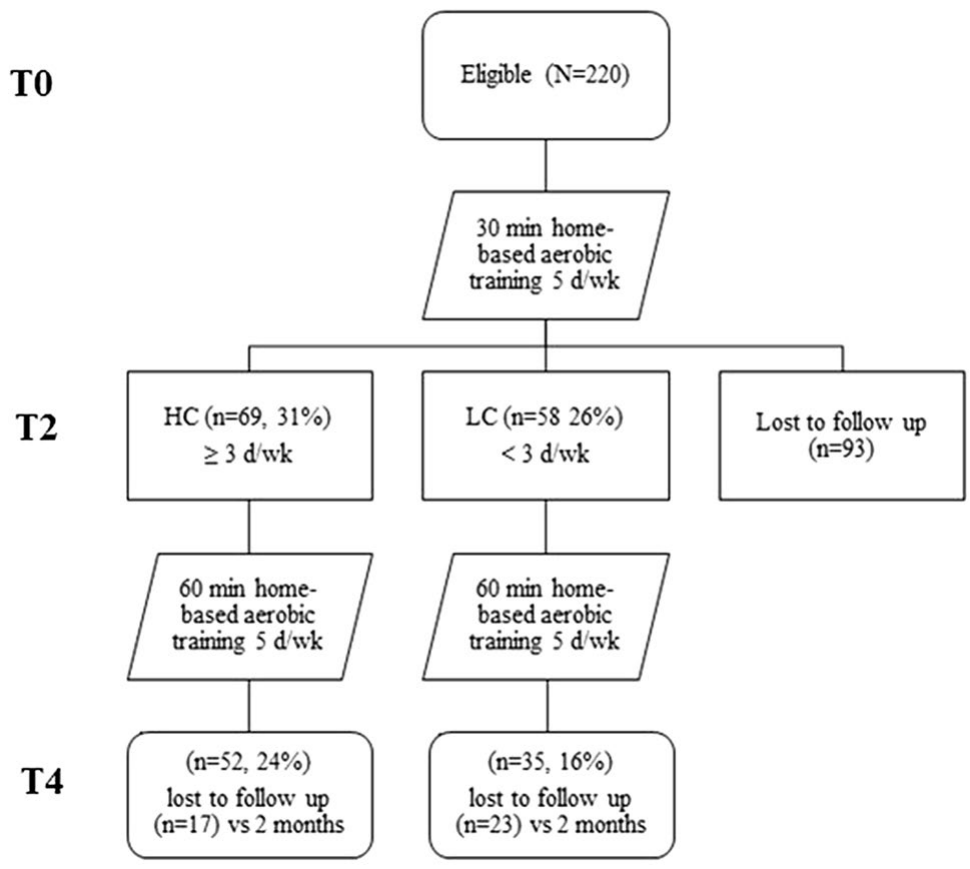
A schematic diagram indicating the flow of study subject selection through the study and subject compliance. *T0* baseline, *T2* after 2 months, *T4* after 4 months, *HC* high compliance (≥ 3 times/week), *LC* low compliance (< 3 times/week)

We studied the outcomes of a 4-month unsupervised home-based aerobic training, in addition to diet program, in obese adults. All participants received an individualized aerobic training in addition to a low calories diet as described in dietetic and physical activity program paragraph. A schematic map of the study design is shown in Fig. 1.

After basal clinical examination, subjects performed a series of tests to analyze body composition, physiological and functional capacities. Body composition and functional evaluation measurements were performed at the Department of Movement, Human and Health Sciences at the University of Rome “Foro Italico”.

During the intervention period, all subjects received an individualized hypocaloric diet and exercise programs. Six-minute walking test (6MWT) was performed to assess individual functional capacity [25]. Then a metabolic incremental test on a treadmill was performed to assess cardiorespiratory capacity and IVT. All subjects were tested in the morning from 9.00 am to 12 pm. The exercise program consisted in 4 months of aerobic training based on a HR, walking speed, and OMNI-RPE-Walk/Run score corresponding to the IVT. All subjects were requested to fill out a daily physical activity diary. Our study cohort consisted of the 87 (40%) subjects who completed the program. According to the compliance to exercise program, subjects were divided in two groups: high compliance (HC, exercise performed 3–5 days/week), and low compliance (LC, exercise performed < 3 days/week).

### Body composition

Pre- and post-intervention anthropometric measurements assessed subjects’ weight, height, body mass index (BMI), percentage of fat mass (% FM) and fat-free mass in kilograms (FFM). Weight and height were measured using a scale and a stadiometer to the nearest 0.1 kg and 0.1 cm, respectively. BMI was calculated as ratio between weight and square of the height (kg/m^2^). Moreover, for each subject, % FM and FFM were measured by hand–foot bioelectrical impedance method (Tanita BC 601, Tokyo, Japan) [24] while the subjects wore minimal clothing (i.e., underwear).

### Physiological capacity

Each subject rested quietly for 5 min to assess the resting HR. The mean HR of the last minute was taken as the resting value.

The peak oxygen uptake ($\dot{V}$O_2peak_) was assessed in all participants by means of a continuous, incremental graded exercise test on a treadmill (Woodway PRO, Woodway, Waukesha, WI, USA). $\dot{V}$O_2_, carbon dioxide production ($\dot{V}$CO_2_), and $\dot{V}$E were measured on a breath by breath basis by a gas analysis system (Quark RMR-CPET Cosmed™, Rome, Italy) [26]. The system was calibrated before each test according to the manufacturer’s instructions.

The continuous graded incremental treadmill protocol started at 3 km/h, then the speed was increased by 1 km/h every 2 min until 5 km/h was reached. Then, slope was increased by 3% every 2 min until one of the following conditions was reached: a value of 10 on RPE-OMNI-Walk/Run Scale [22] or the subject’s HR reached a value of 90% of their HR_max_. HR (beats/min) was continuously recorded before and throughout the trial using a HR monitor (RS 400, Polar Electro™, Kempele, Finland) [12].

During the test, the highest $\dot{V}$O_2_ attained was chosen as the $\dot{V}$O_2peak_. The individual ventilatory threshold (IVT) was determined offline for each subject by plotting the ventilatory equivalent ($\dot{V}$E/$\dot{V}$O_2_) as a function of $\dot{V}$O_2_ to identify the point during exercise where this curve reached its lowest value [27]. The level of $\dot{V}$O_2_ at which we observed the lowest value of the $\dot{V}$E/$\dot{V}$O_2_, in the individual plot, was the individual ventilatory threshold [11, 12, 27]. The exercise intensity corresponding to IVT was reached by all the subjects. At IVT and at maximal effort, HR and metabolic equivalent (MET) were determined. In addition, % HR_max_ [(HR at IVT)/(220-age)] was also calculated.

A standard definition of OMNI-Walk/Run Scale was explained to the subjects immediately before the exercise test. During exercise testing, subjects were asked to report the degree of exertion on the OMNI-Walk/Run scale every 2 min. The OMNI-Walk/Run scale illustration was in full view of the subject at all times during testing.

### Functional capacity

The *6*-*min walking test* measures the distance covered by subjects in 6 min [25]. Briefly, subjects were instructed to walk as fast as they could in a 50-m corridor marked every 5 m with white tapes on the floor. Subjects were allowed to stop or rest during the test if necessary. Standardized encouragements were provided at recommended intervals.

Distance walked (6MWT_dist_) and subjects’ HR_mean_ during the 6-min walking test were measured, speed (6MWT_speed_) was calculated (m/s).

### Dietetic and physical activity program

A hypocaloric diet was set at approximately 400 kcal less than total daily energy expenditure as described elsewhere (manuscript in preparation) with nutrients subdivided on the base of Mediterranean Diet scheme. Total daily energy expenditure was determined by adding the resting metabolic rate and the physical activity level. Resting metabolic rate was estimated by the Cunningham equation [28] while physical activity level was estimated by the international physical activity questionnaire (IPAQ) [29].

All subjects were requested to perform 4 months of unsupervised aerobic training, minimum five times a week, based on HR, walking speed, and on the RPE-OMNI-Walk/Run score corresponding to their IVT as prescribed on basal evaluation. The training session duration varied between 30 min in the first 2 months and 60 min during the following 2 months. Each subject filled out a daily physical activity diary including track length, duration, HR, and RPE-OMNI over the entire training period. After 2 months of intervention, subjects were re-evaluated to prescribe a new workload, leading to maintenance of the individual moderate exercise intensity constant during the 4-month period. Also, the volume of exercise was increased reaching a maximum of 60 min per daily session.

### Statistical analysis

All results were expressed as mean ± SD. At the beginning of the study, differences on body composition between males and females were evaluated with a multiple unpaired *t* test with Bonferroni correction. For each variable, a mixed 2×3 ANCOVA with repeated measures on time was used to detect significant effects of two main factors: group (high compliance vs. low compliance) and time (pre vs. 2 vs. 4 months). Since differences were present in male and female population, age, gender and height were used as covariates.

Significant interactions were further analyzed by means of appropriate post hoc analysis. All statistical analyses were performed with the SPSS statistical package (Version 24.0 for Windows; SPSS Inc., Chicago, IL, USA). All tests were two-tailed, with *p* ≤ 0.05 being taken as significant.

## Results

Evaluation of adherence to physical activity training protocol in the study population, 43% subjects dropped out after 2-month training, while 31% had high adherence and 26% had low adherence. At the end of the 4-month training 60% subjects dropped out, while 24% had HC and 16% had LC. All subjects were contacted by telephone to understand the reasons of drop-out. Lack of time followed by health status and clinical condition factors have been reported to limit participation to the protocol. No subjects got injured during the training protocol.

### Body composition variables

**Fig. 2:**
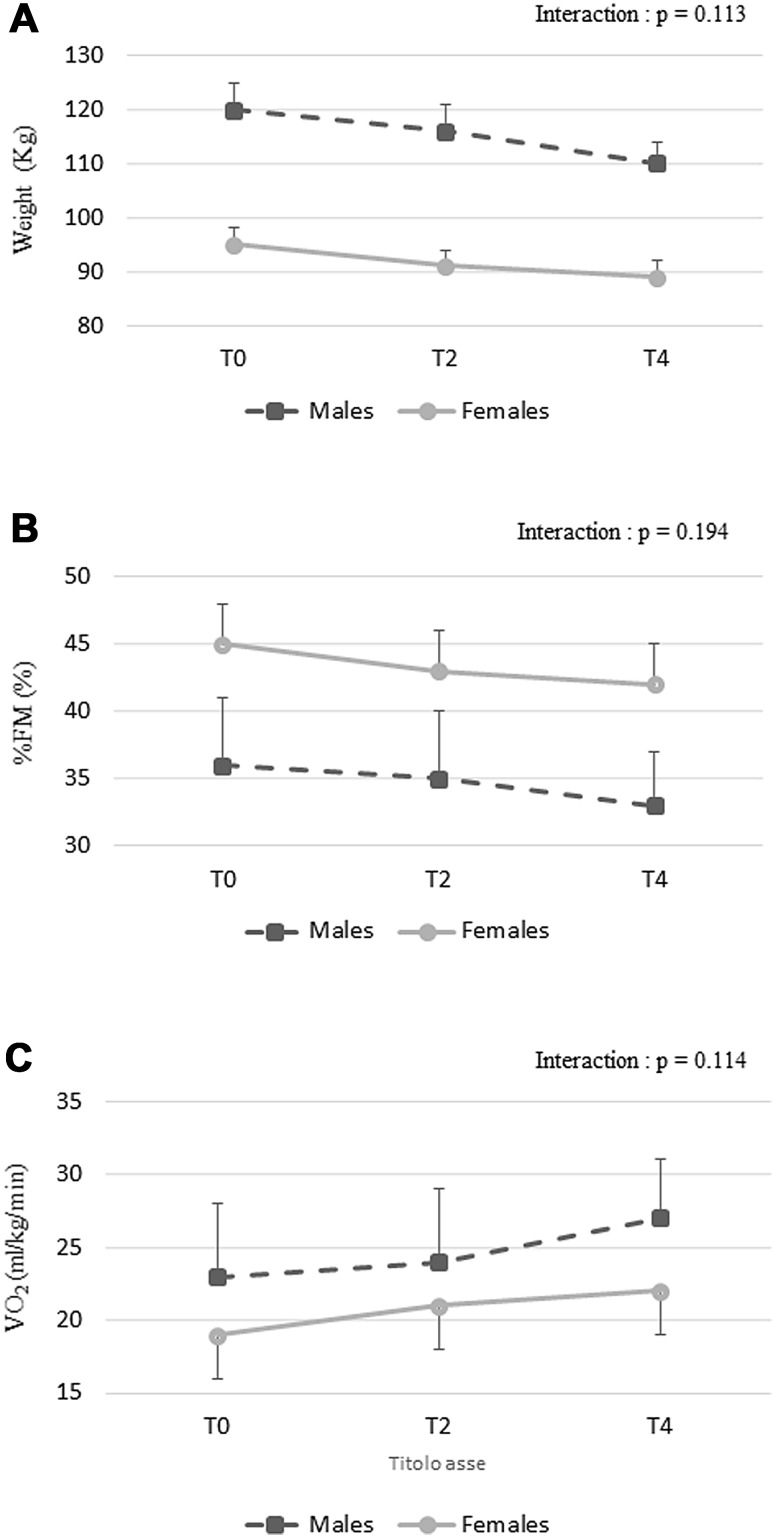
Time × gender interaction for weight (**a**), % FM (**b**), and $\dot{V}$O_2_ at peak (**c** variables during training intervention. *T0* baseline, *T2* after 2 months, *T4* after 4 months. Data are presented as mean ± SD

Anthropometric characteristics of female and male subjects who performed all training are depicted in Table 2. Significant main effect of time (*p* < 0.01) and group × time interactions (*p* < 0.001) were found for weight, BMI, and FM %. Post hoc analysis showed that weight, BMI, and % FM significantly decreased after 2 and 4 months of training in HC group. No significant changes were found in LC group (Table 2). No significant time × gender interaction was found for body composition variable (Fig. 2a, b).

**Table 2:** Subjects’ anthropometric parameters at baseline (T0) and after 2 (T2) and 4 (T4) months of intervention

	HC (*n* = 52)			LC (*n* = 35)		
	T0	T2	T4	T0	T2	T4
Weight (kg)	101.3 ± 21.4	97.31 ± 20.5**	94.2 ± 18.3**^a^	95.1 ± 20.7	94.7 ± 20.7	95.7 ± 20.8
BMI (kg/m^2^)	37.6 ± 6.5	36.1 ± 6.4**	34.9 ± 5.7**^a^	35.4 ± 5.7	35.3 ± 5.6	35.6 ± 5.6
FFM (kg)	59.2 ± 12.6	59.3 ± 12.9	58.5 ± 12.5	54.4 ± 12.6	55.4 ± 14.0	55.0 ± 12.5
FFM (%)	57.3 ± 7.9	59.0 ± 6.7**	59.9 ± 7.2**^b^	57.6 ± 5.6	58.3 ± 6.1	57.5 ± 6.2
FM (%)	42.7 ± 6.5	41.0 ± 6.3**	40.1 ± 6.8**^a^	42.4 ± 5.4	41.7 ± 5.8	42.5 ± 5.8

*BMI* body mass index, *FFM* fat-free mass, *FM* fat mass

**p < 0.01 vs. T0

^a^ *p* < 0.01 vs. T2

^b^ *p* < 0.05 vs. T2

### Physiological capacity

A significant main effect of time was observed for $\dot{V}$O_2peak_ (*p* = 0.02) and MET_peak_ (*p* = 0.02) during the incremental treadmill test. Interestingly, no significant time × gender interaction was found for $\dot{V}$O_2peak_ variable (Fig. 2c). A significant group × time interaction was found for $\dot{V}$O_2peak_ (*p* = 0.02) and MET_peak_ (*p* = 0.02). Post hoc analysis showed that $\dot{V}$O_2peak_, and MET_peak_ significantly increased after 2 and 4 months of training in HC group while no significant changes were found in LC group (Table 3).

**Table 3:** Adjusted parameters at peak during the incremental treadmill test

	HC (*n* = 52)			LC (*n* = 35)		
	T0	T2	T4	T0	T2	T4
$\dot{V}$O_2_ peak (ml/kg/min)	20.3 ± 4.1	21.9 ± 4.1*	22.9 ± 5.1**	21.2 ± 3.9	21.1 ± 4.4	21.6 ± 3.1
Ab$\dot{V}$O_2_ (ml/min)	2103.5 ± 513.3	2088.6 ± 545.4	2117.3 ± 611.3	2004.8 ± 519.2	1991.3 ± 546.0	2052.9 ± 473.8
MET peak	5.8 ± 1.2	6.3 ± 1.2*	6.5 ± 1.5**	6.1 ± 1.5	6.0 ± 1.5	6.2 ± 1.0
HR (bpm)	142.5 ± 16.5	143.0 ± 16.3	141.3 ± 20.6	146.2 ± 17.1	140.9 ± 17.1	145.4 ± 18.3
% HR_max_ (%)	83.8 ± 8.2	84.0 ± 7.1	82.9 ± 9.9	85.1 ± 8.1	82.0 ± 7.5	84.6 ± 8.1

Data at baseline (T0) and after 2 (T2) and 4 (T4) months of intervention

$\dot{V}$ *O*_*2*_ oxygen uptake, *Ab* $\dot{V}$ *O*_*2*_ oxygen uptake in ml/min, *MET* metabolic equivalent, *HR* heart rate, *% HR*_*max*_ heart rate expressed as percentage of HR_max_ (220-age)

**p < 0.01 vs. T0

*p < 0.05 vs. T0

No significant main effect of time and group and no significant group × time interaction were found for any parameter at IVT during the incremental treadmill test (Table 4).

**Table 4:** Adjusted parameters at individual ventilatory threshold (IVT) during the incremental treadmill test

	HC (*n* = 52)			LC (*n* = 35)		
	T0	T2	T4	T0	T2	T4
$\dot{V}$O_2_ (ml/kg/min)	14.8 ± 4.2	14.8 ± 3.1	15.8 ± 5.0	15.0 ± 1.9	14.5 ± 2.8	14.8 ± 2.6
Ab $\dot{V}$O_2_ (ml/min)	1462.2 ± 540.7	1392.8 ± 362.9	1448.9 ± 519.2	1465.5 ± 332.5	1402.6 ± 328.2	1432.7 ± 278.2
% $\dot{V}$O_2peak_ (%)	73.3 ± 17.5	67.9 ± 10.2	69.1 ± 12.7	71.2 ± 15.1	66.7 ± 11.5	66.6 ± 12.1
MET	4.2 ± 1.2	4.2 ± 0.9	4.5 ± 1.4	4.3 ± 0.5	4.1 ± 0.8	4.2 ± 0.7
HR (bpm)	117.9 ± 15.6	114.0 ± 12.3	114.5 ± 16.5	122.8 ± 13.1	119.8 ± 11.6	121.5 ± 11.6
% HR_peak_ (%)	69.4 ± 8.9	67.0 ± 5.9	67.3 ± 8.6	70.6 ± 9.9	68.7 ± 7.0	69.8 ± 8.3
RPE	3.6 ± 2.5	3.4 ± 2.0	3.4 ± 2.0	3.6 ± 2.5	3.8 ± 2.0	3.7 ± 2.0

Data at baseline (T0) and after 2 (T2) and 4 (T4) months of intervention

*%* $\dot{V}$ *O*_*2peak*_ oxygen uptake expressed as percentage of $\dot{V}$O_2peak_, *MET* metabolic equivalent, *HR* heart rate, *% HR*_*max*_ heart rate expressed as percentage of HR_max_ (220-age), *RPE* OMNI scale

### Functional capacity test

No significant main effect of time and group was found for speed, distance, and HR mean during the 6MWT. A significant group × time interaction was found for 6MTW_dist_ (*p* = 0.04), 6MWT_speed_ (*p* = 0.04), and HR_mean_ (*p* < 0.01). Post hoc analysis demonstrated that 6MTW_dist_ and 6MWT_speed_ significantly increased while HR_mean_ significantly decreased after 4 months of training in HC group. In contrast, no significant changes were found in LC group (Table 5).

**Table 5:** Adjusted six-minute walking test (6MWT) parameters at baseline (T0) and after 2 (T2) and 4 (T4) months of intervention

	HC (*n* = 52)			LG (*n* = 35)		
	T0	T2	T4	T0	T2	T4
6MWT_dist_ (m)	567.9 ± 76.4	590.1 ± 59.8**	604.8 ± 69.1**^a^	590.7 ± 67.2	587.5 ± 61.7	597.2 ± 58.7
6MWT_speed_ (m/s)	1.58 ± 0.22	1.65 ± 0.17**	1.67 ± 0.19**^a^	1.65 ± 0.18	1.63 ± 0.18	1.66 ± 0.16
HR_mean_ (bpm)	131.7 ± 18.7	126.7 ± 16.4*	123.9 ± 15.3**	130.9 ± 18.0	131.5 ± 15.6	131.4 ± 15.6
RPE	5.1 ± 2.3	5.0 ± 2.0	4.7 ± 1.7	6.3 ± 1.8	6.0 ± 1.7	5.8 ± 1.8

*6MWT*_*dist*_ distance walked during the 6-min walking test, *6MWT*_*speed*_ subjects’ speed during the 6-min walking test, *HR*_*mean*_ mean heart rate during the 6-min walking test

**p < 0.01 vs. T0

*p < 0.05 vs. T0

^a^ *p* < 0.05 vs. T2

## Discussion

The results presented in the study demonstrate that the use of walking speed, HR and, OMNI-Walk/Run scale variables, corresponding to the IVT, leads to an improvement of body composition and physiological variables in obese subject independently from genders.

Aims of this study were to evaluate (1) adherence and (2) effects of 4-month home-based unsupervised aerobic training, based upon the HR, walking speed, and RPE-OMNI score corresponding to the IVT, on body composition, physiological and physical capacities in female and male obese adults.

The drop-out rate (60%) after 4-month training, even if the exercise intensity was individualized according to subject’s health, confirms that the incorporation of training into everyday life is still challenging in obese population without gender differences. Graffagnino et al. [7] reported that after the 6-month supervised weight loss program, offered in a community-based medical wellness facility, the drop-out rate was 53%. In contrast, Arikawa et al. [30] showed that using strategies based on social cognitive theory, women’s adherence to a 4-month supervised weight training intervention was higher than 90% during the training period. In our study, strategies based on social cognitive theory, such as incentive, phone and email reminders and personal booster sessions were not employed to encourage and support adherence. Our experience indicate that the promotion of physical activity is more convincing when the intensity of exercise proposed is low rather than high [8, 23]. Thus, in a daily routine training program, other parameters, such as social and cognitive factors, demographic factors, and environmental factors, should be taken into considerations beside exercise intensity [30]. A very interesting point was to assess that up to 40% of subjects that completed the 4-month period, only 24% had HC to exercise while 16% had LC. This 16% of subjects did not fulfill the physical recommendation but they were still on the program. This could be seen as a positive result, since, with the introduction of motivation factors, it might be possible to increase the compliance to the program. On the basis of the results obtained and described in this manuscript, we are planning training protocols in obese subjects with the aim to analyze differences on psychological and cognitive factors between subjects with high or low compliance to the training program.

The results obtained only in HC group of the present study suggest that the use of this new approach could be a valuable tool for physical intervention in obese subjects to improve body composition, physiological and functional capacity. The results of HC group after home-based unsupervised training indicate that this program is as efficient as a supervised frequent walking performed more than 3 days a week [16]. Furthermore, according to Perri et al. [31], middle-aged sedentary individuals, particularly those who are overweight and unfit, might be particularly susceptible to interrupt an activity that entails a relatively high degree of subjective discomfort. For this reason, our study proposed an individualized intensity corresponding to moderate exercise that was appropriate for obese subjects. Interestingly, our results also suggest that no gender differences are present in obese individuals in term of all parameters evaluated in the study. This is an important point, since it might indicate that life style correction positively influences physical parameters in both gender, which also suggest that a physical activity protocol designed on individual aerobic threshold may induce similar adaptations in male and female obese subjects.

Inducing negative energy balance is the most important goal of weight loss programs and exercise plays a pivotal role to imbalance the energy equation to obtain weight loss [32]. An undesirable effect could be that weight loss program might induce a concomitant decline in lean tissue. Regarding the effects on body composition, the body weight and BMI decreased significantly as well as FM % in HC group. Moreover, fat-free mass expressed in kg was maintained after a mix of training and diet. All these results highlight how an integrated approach of diet and physical activity can elicit a considerable general improvement of body composition in obese subjects. These results are in accordance with other studies [11, 32] that verified the positive effects of walking exercise to contrast the decline of lean mass. Moreover, even though LC group received a calorie restricted diet, no differences were found in any body composition variables between pre- and post-intervention. This result could be explained by a low adherence to a hypocaloric diet, beside the low adherence to the physical activity program during the study. Exercise alone has not been shown to produce substantial weight loss, but it is helpful during the weight loss phase to preserve lean muscle mass, and it plays a role in body weight maintenance [33, 34]. We might affirm that exercise intensities based upon heart rate, walking speed, and RPE-OMNI score at IVT play positive effects in maintaining FFM during a weight loss program in HC female and male obese subjects. These findings are significant, given that FFM represents a key determinant of the magnitude of resting metabolic rate (RMR). It follows that a decrease in lean tissue could hinder the progress of weight loss.

Even if the mix of aerobic and resistance training might have higher impact on body composition, in this study we did not prescribe resistance training since it is very difficult to perform this type of exercise in an unsupervised home-base settings due to the lack of specific devices, such us bench press, leg press, free weights, and the risk of performing exercises in a wrong manner.

Regarding the peak exercise capacity of these subjects, data showed that they were untrained. Before intervention $\dot{V}$O_2peak_ was 20.2 and 19.3 ml/kg/min in HC and LC group, respectively. Moreover, during incremental exercise testing subjects did not reach their HR_max_ (220-age) but they stopped around 83% of their maximal HR. Therefore, if exercise intensity is prescribed according only to relative parameters, based upon HR_max_, without individual adjustment on exercise capacity, the exercise intensity could be overestimated in obese subjects of both genders. Before the training program, the subjects $\dot{V}$O_2peak_ is in agreement with the literature regarding the physical fitness in obese subjects [35, 36]. Conversely, the cardiorespiratory fitness reflected by the absolute $\dot{V}$O_2peak_ (ml/min) is still preserved as reported for obese women of similar age [36]. After aerobic training HC group had significantly higher $\dot{V}$O_2peak_ and MET than at baseline. These results could be justified by the positive effects of moderate intensity exercise on weight loss. In the current study, $\dot{V}$O_2peak_, increased by 11% during a 4-month intervention and this result was similar to those reported in another study, which showed an increase of 9.5% in a group of 15 obese subjects with kidney disease who submitted to home-based aerobic training according to IVT [15]. Aoike et al. [15] showed that a home-based exercise program based upon HR, corresponding to IVT, was safe and effective in improving cardiopulmonary and functional capacities of overweight patients with CKD. However, this study did not analyze the dropout rate and did not use RPE and waking speed, corresponding to IVT, as training variables. Our results, regarding a 4-month unsupervised aerobic training, are in agreement with those reported by Belli et al. [16], who proposed the ventilatory threshold as parameter to conduct 12 weeks of supervised aerobic training intervention in type 2 diabetic women. The authors recruited a small sample of participants (IG = 9; CG = 10) obtaining improvements in body weight (75.0 ± 6.1; 72.5 ± 5.9 kg), fat mass (30.6 ± 4.2; 27.2 ± 3.4 kg), BMI (32.2 ± 2.0; 31.1 ± 1.9), and body fat (39.5 ± 2.4; 36.6 ± 1.9%). Moreover, no significant change was found in fat-free mass (44.4 ± 1.9; 45.4 ± 2.8 kg), suggesting that fat-free mass might not be lost during an aerobic training if a programmed and individualized resistance training effort is not added. In particular, we chose to use unsupervised aerobic training to allow obese individuals to perform physical activity as a daily workout routine. Seeing that $\dot{V}$O_2peak_ increased after training and no differences were assessed for any variables at IVT, after training, we might speculate that the capacity of obese subjects to perform exercise above the IVT was increased.

In conclusion, our results showed that only HC increased 6MWT distance and speed. Moreover, HR_mean_ during 6MWT was lower after training than pre training. These positive results highlight that subjects’ fitness level increased after aerobic training. In fact, subjects were able to cover more distance with an HR lower than the pre training. Before training, the mean distance covered during 6MWT was 585 m, which is comparable with a previous study in obese who walked 548 m [37].

### Study limitations

We are aware that our physical performance evaluation did not assess all fitness parameters (e.g., strength and flexibility). Moreover, we are aware that our evaluation also lacks hormonal parameters that might be modified after training period and consequently modify the body composition and physiological parameters. Indeed, we believe this is an important point to be evaluated to understand and characterize the mechanisms underlying the improvement of body composition obtained by the obese subjects upon physical and nutritional protocol intervention and, thus, we are in the process to further expand our study, also increasing the number of subjects, to fully address these specific points.

However, due to the great impact of morbid obesity on these subjects, we tried to implement the most important baseline standards to be performed by this population. Further studies are needed to evaluate different protocols that include both resistance and aerobic training. Moreover, we were not able to control whether the participants were adherent to the prescribed diet during the study. The lack to diet adherence could have attenuated the results on both body weight and body composition.

This is the first study that characterizes the use of heart rate, speed, and OMNI-RPE score variables, corresponding to individual IVT, as useful parameters for home-based unsupervised aerobic training to increase physical fitness in female and male obese adults. This method, without strategies based on social cognitive theory, results in a 60% of drop-out rate within 4-month of training with 24% of subjects that had HC to exercise.

The present study suggests that an individualized home-based aerobic training based upon IVT might positively influence the individuals’ lifestyle. Our results also indicate that the use of walking speed, HR and, OMNI-Walk/Run scale variables, corresponding to the IVT, might be a useful tool to prescribe home-based moderate aerobic training in obese subjects. However, this method should be implemented with psychological and cognitive strategies to increase the number of patients who can introduce physical activity in a daily routine.
